# Molecular evidence of *Ehrlichia canis* and *Anaplasma platys* and the association of infections with hematological responses in naturally infected dogs in Kalasin, Thailand

**DOI:** 10.14202/vetworld.2019.131-135

**Published:** 2019-01-23

**Authors:** Supawadee Piratae, Priyakorn Senawong, Pornchalerm Chalermchat, Warissara Harnarsa, Benjawan Sae-chue

**Affiliations:** 1 Office of Academic Affairs, Faculty of Veterinary Sciences, Mahasarakham University, Maha Sarakham, Thailand; 2 One Health Research Unit, Faculty of Veterinary Sciences, Mahasarakham University, Maha Sarakham, Thailand; 3 Department of Infectious Disease Control, Faculty of Medicine, Oita University, Oita, Japan

**Keywords:** packed cell volume, platelet count, Thailand, tick-borne pathogens

## Abstract

**Background:**

Tick-borne bacteria, *Anaplasma platys* and *Ehrlichia canis* are well recognized as the etiology of anemia and thrombocytopenia in dogs. The clinical signs of anaplasmosis and ehrlichiosis range from asymptomatic to severe symptoms . There are insufficient studies about epidemiological surveys of these blood parasites, also the association of infections with the hematological study.

**Aim:**

This study aimed to screen *A. platys* and *E. canis* in naturally infected dogs and the effects of the infection on the levels of packed cell volume (PCV) and platelet count.

**Materials and Methods:**

A total of 68 blood samples were collected from free-roaming dogs at Nong Kung Sri district, Kalasin Province, Thailand, and examined for *A. platys* and *E. canis* infection by polymerase chain reaction (PCR) and measured PCV levels and platelet count.

**Results:**

Using nested PCR, 42.65% of dogs were infected with one or two pathogens. The molecular detection of anaplasmosis and ehrlichiosis in this population was 29.4% (95% confidence interval [CI]: 18.98-41.71) and 25% (95% CI: 14.4-35.3), respectively. Coinfection occurred at 11.8% (95% CI: 5.22-21.87). Infection with *E. canis* and coinfection showed significant association with PCV levels (p<0.05) while *A. platys* infection showed no statistical relationship. Infection with *A. platys*, *E. canis*, and coinfection had a non-significant correlation with platelet count (p>0.05).

**Conclusion:**

This study provides data of anaplasmosis and ehrlichiosis in free-roaming dogs which indicated that these zoonotic diseases are widespread and require for disease frequency determination, especially in Kalasin Province of Thailand where data of tick-borne infections in dogs have not been reported.

## Introduction

Canine ehrlichiosis and anaplasmosis are the infectious diseases caused by bacteria of the order Rickettsiales, family Anaplasmataceae, and genera *Ehrlichia* (ehrlichiosis) and *Anaplasma* (anaplasmosis) that are transmitted by ixodid ticks [1]. In Thailand, *Ehrlichia canis* and *Anaplasma platys* infections are commonly diagnosed [2,3] which the principal vector of these parasites is the brown dog tick, *Rhipicephalus sanguineus* [4]. Dogs acquire infections through the bite of infected ticks. These tick-borne diseases remain veterinary relevance in tropical and subtropical countries due to the spread of the vector and agents, furthermore, are increasingly recognized as zoonosis [5,6].

Mostly of *E. canis* develops within the monocytes caused canine monocytic ehrlichiosis (CME). Clinical signs of CME are variation depend on stages of disease (acute phase, subclinical, and chronic) which may be obvious by fever, depression, lethargy, anorexia, lymphadenomegaly, splenomegaly, anemia, leukopenia, and thrombocytopenia [7,8]. For *A. platys*, the target cells of infection are platelets which cause canine cyclic thrombocytopenia. Although infected dogs are mild-to-moderate clinical signs, high severity will be happened in multiple infections with others pathogens, especially coinfections with *E. canis* which can lead to severe thrombocytopenia [9].

Parasite diagnosis has usually performed by blood smear examination based on parasites morphology which is easy and not expensive but less sensitivity in case of low bacteremia and less specificity in case of low experience examiner. Serological is the alternative ways to investigations but cannot distinguish the presence antibody whether real-time infections or past infection. The molecular method by polymerase chain reaction (PCR) is now common and widely used in laboratories allows for high specificity and sensitivity although low bacteremia or early stage of infection [10].

The objectives of this study were to determine *A*. *platys* and *E. canis* infections by PCR and to analyze the association between pathogens infections with packed cell volume (PCV) (%) and platelet count in naturally infected dogs from an endemic area in Kalasin, Thailand.

## Materials and Methods

### Ethical approval

All experimental procedures involving animals were conducted in accordance to Animals for Scientific Purpose Act B.E. 2558 (A.D. 2015) (U1-01509-2558) and approved by the Institutional Animal Care and Use Committee, Mahasarakham University (0031/2017).

### Sample collection

This cross-sectional was surveyed on August 2017, 68 blood samples were collected from free-roaming dogs at Nong Kung Sri district, Kalasin Province, Thailand. In this study, we collected blood samples from free-roaming owned dogs which are able to roam freely. Blood was collected approximately 1-2 ml from the cephalic vein into sterile tubes with ethylenediaminetetraacetic acid (EDTA) anticoagulant and kept on ice during transport to the laboratory. The remaining blood after PCV level determinations and platelet measurements was stored at –20°C until DNA extraction for long-term preservation. All steps for animal handling and blood collections were conducted by veterinarians, and all dogs were approved from their owners.

### Measurement of PCV levels

Levels of the PCV were estimated by fill blood directly into 2/3 of the heparinized microhematocrit tube. Place the tube into a calibrated microhematocrit centrifuge and spin at 10,000 rpm. Measuring the height of the total blood column and the height of the red cell layer within a minute after the centrifuge has stopped. PCV levels were classified according to anemia severity as follows: ≥37%, 30-37%, 20-29%, and ≤20% are non-anemia, mild anemia, moderate anemia, and severe anemia, respectively [11].

### Measurement of platelet count

Levels of the platelet count were evaluated by thin blood smear method. Drop the blood (10-20 µl) on to a clean slide then spreads the drop of blood with a clean spreader slide. Until the blood smear slides are completely dry, fix with methanol 2 min, and stain with Giemsa, respectively. Blood films were observed under a light microscope for platelet counting. The platelet count grades were classified into four groups according to thrombocytopenia severity as follows: Non-thrombocytopenia (platelet ≥200,000 cell/µl), mild thrombocytopenia (platelet ≤200,000 cell/µl), moderate thrombocytopenia (platelet ≤150,000 cell/µl), and severe thrombocytopenia (platelet ≤100,000 cell/µl).

### DNA extraction and amplification of tick-borne pathogens

About 200 µl of EDTA anticoagulated whole blood were used for DNA extraction using GF-1 blood DNA extraction kit (Vivantis). Concentrations of total DNA were determined by exposing the DNA to ultraviolet light at a wavelength of 260 nm with NanoDrop 1000 Spectrophotometer (Thermo Scientific). *E. canis* and *A. platys* 16srRNA gene was amplified by nested PCR. In amplification steps, primers used in PCR for the detection of DNA of *A. platys* and *E. canis* were selected based on previously reported. The first step of amplification used universal primers for rickettsia (ECC 5’ AGA-ACG-AAC-GCT-GGC-GGC-AAG-CC 3’, ECB 5’ CGT-ATT-ACC-GCG-GCT-GCT-GGC-A 3’) and the second step used *E. canis* specific primer (CANIS 5’ CAA-TTA-TTT-ATA-GCC-TCT-GGC-TAT-AGG-A 3’, HE3 5’ TAT-AGG-TAC-CGT-CAT-TAT-CTT-CCC-TAT 3’) and *A. platys* specific primer (PLATYS 5’ TTT-GTC-GTA-GCT-TGC-TAT-G 3’, GA1UR 5’ GAG-TTT-GCC-GGG-ACT-TCT-TCT 3’) [12-15].

PCR contained approximately 50 ng of extracting DNA, 10 pmol of each primer, 200 µM of each dNTPs, 1.5 mM of MgCl_2_, and 1 unit *Taq* polymerase (Vivantis). PCR protocols were done with 35 cycles of denaturation at 95°C for 45 s, annealing at 60°C and 62°C for the 1^st^ and 2^nd^ steps of *A. platys* and 60°C for both two steps of *E. canis* for 45 s, extension at 72°C for 90 s, and a final extension at 72°C for 5 min. PCR amplification was performed using Biometra GmbH thermocycler (Germany). PCR products were identified by 1% agarose gels stained with ethidium bromide and visualized under ultraviolet light.

### Statistical analysis

Sample size was calculated followed [16] with the prevalence of 23% [17] error 0.1 and α=0.05. The prevalence (%) and 95% confidence intervals (%CI) were calculated using descriptive statistics by online software (http://sampsize.sourceforge.net/iface/index.html). The association between blood pathogens infection with tick infestation, level of PCV, or platelet count was compared with Pearson’s Chi-squared test.

## Results and Discussion

### Dog characteristics and molecular detection of *A. platys* and *E. canis*

Of the 68 blood samples, 44 (64.7%) were collected from male dogs and 24 (35.3%) from female dogs, aged between 5 months and 14 years with 9 (13.2%) from young dogs (≤12 months) and 59 (86.8%) from adult dogs. Simple physical examination revealed that most dogs were regular and 98.5% of dogs were no abnormalities seen in hydration status. The prevalence of infections in male dogs was 50% (22/44), and female dogs was 29.17% (7/24). Tick infestation (nymph, engorge adults, and non-engorge adults) individual was found in 50% of dogs. Addition of those which are infected dogs (29/68), 16 cases were infested with ticks, and 13 cases were not found ticks in their bodies (Table-1). Statistical analysis from Pearson’s Chi-squared test revealed no different infections between found ticks group and not found ticks group (p=0.462). Some ticks may fail to be vector of transmission due to dogs were clinically healthy, and the amount of circulating pathogens was not enough to be transmitted to ticks [18].

**Table-1:** Prevalence of *A. platys* and *E. canis* infections by demographic characteristics of dogs.

Characteristics	Number of dogs (%)	Number of infected dogs (n=29)					

		A	CI (%)	E	CI (%)	A+E	CI (%)
Sex (n=68)							
Male	44 (64.7)	16	36.4 (22.41-52.23)	13	29.5 (16.76-45.20)	7	15.9 (6.64-30.07)
Female	24 (35.3)	4	16.7 (4.74-37.38)	4	16.7 (4.74-37.38)	1	4.2 (0.11-21.12)
Age (n=68)							
≤1 year	9 (13.2)	3	33.3 (7.49-70.07)	1	11.1 (0.28-48.25)	1	11.1 (0.28-48.25)
1-14 years	59 (86.8)	17	28.8 (17.76-42.08)	16	27.1 (16.36-40.27)	7	11.9 (4.91-22.93)
Ticks (n=68)							
Found	34 (50)	10	29.4 (15.10-47.48)	11	32.4 (17.39-50.53)	5	14.7 (4.95-31.06)
Not found	34 (50)	10	29.4 (15.10-47.48)	6	17.7 (6.76-34.53)	3	8.8 (1.86-23.68)
Positive							
Single and coinfection	29 (42.65)	20	29.4 (18.98-41.71)	17	25.0 (14.4-35.3)	8	11.8 (5.22-21.87)
Single infection	21 (30.9)	12	17.7 (9.46-28.80)	9	13.2 (6.23-23.64)		

*A. platys=Anaplasma platys, E. canis=Ehrlichia canis*, CI (%)=95% Confidence interval

To the authors’ knowledge, this study is the first report of the molecular prevalence of *A. platys* and *E. canis* in dogs in Kalasin, Thailand. The prevalence of anaplasmosis and ehrlichiosis was 29.4% and 25%, respectively. Coinfection occurred at 11.8% (Table-1). In Thailand, the percentage of *A. platys* is similar to that described in Maha Sarakham Province (29.2%) [11], but different from which reported in Songkhla (4.4%) [19], correlation with *E. canis* infection rate that is comparable to that described in Maha Sarakham Province (21.5%) [17], but dissimilar from which reported in Songkhla (3.9) [19]. However, these results might be explained by locality, where Maha Sarakham and Kalasin are adjoining provinces in the Northeastern part of Thailand may be similar in structure, climate, and humidity. In other regions, the prevalence of tick-borne pathogens in dogs is variation depended on sampling locality, spreading of the tick vector, the opportunity of hosts to expose with infected ticks, season [20], and parasitic examination techniques. For examples, they reported 6% of infections with both *A. platys* and *E. canis* in the Thrace Region of Turkey [21], 34.6% for *A. platys* and 3.3% for *E. canis* in Maio Island of Cape Verde Archipelago [22], 32.9% for *A. platys* in Paraná, Brazil [23], 22.9% for *E. canis* in South West Nigeria [24], etc.

### PCV levels and platelet counts in the dogs infected with *A. platys*, *E. canis*, and coinfection

PCV levels were categorized into four groups; 19, 23, 22, and 4 samples were grouped as non-anemia (>37%), mild anemia (30-37%), moderate anemia (20-29%), and severe anemia (<20%), respectively. Infection with *A. platys* had no significant effect on PCV levels, but infection with *E. canis* and coinfection revealed statistically associated with PCV levels (p<0.05) (Table-2). In addition, Phi correlation in *E. canis*-infected group (r=−0.353) and coinfection group (r=−0.351) showed negative linearly related with PCV levels. The findings of this study support to the previously reported in anemic dogs which showed PCV in *E. canis* infected dogs were significantly lower than the PCV in the non-infected dogs (p<0.05) and also no statistically difference between *A. platys*-infected dogs and PCV levels [25]. Moreover, from our previous study in Maha Sarakham Province, we found the same results which showed *E. canis-* positive dogs presented significant relationship with PCV levels, but dogs infected with *Babesia canis vogeli*, *Hepatozoon canis*, and *A. platys* had no association [11]. However, PCV levels are influenced by various causes including infections with other blood parasites, malnutrition, drug therapy, toxins, or irradiation.

**Table-2:** Association among *E. canis, A. platys*, and coinfections with PCV levels.

Pathogens	Status	PCV levels n (%)				Total Number (%)	p-value	Average PCV value (%)	95% CI of average PCV value (%)

		>37	30-37	20-29	<20				
*E. canis*	+Ve	2 (2.9)	5 (7.4)	10 (14.7)	-	17 (25)	0.037*	29.62	26.47-32.76
	−Ve	17 (25)	18 (26.5)	12 (17.6)	4 (5.9)	51 (75)	-	33.75	31.02-36.47
*A. platys*	+Ve	4 (5.9)	7 (10.3)	9 (13.2)	-	20 (29.4)	0.294	31.90	28.07-35.73
	−Ve	15 (22.1)	16 (23.5)	13 (19.1)	4 (5.9)	48 (70.6)	-	33.05	30.32-35.79
Coinfections	+Ve	-	2 (2.9)	6 (8.8)	-	8 (11.8)	0.039*	26.94	22.99-30.88
	−Ve	19 (27.9)	21 (30.9)	16 (23.5)	4 (5.9)	60 (88.2)	-	33.48	31.08-35.89
Total		19 (27.9)	23 (33.8)	22 (32.4)	4 (5.9)	68 (100)	-	-	-

PCV=Pack cell volume, +Ve=Positive, −Ve=Negative,

* p<0.05, *A. platys=Anaplasma platys, E. canis=Ehrlichia canis*, CI=Confidence interval

In part of thrombocytopenia, we divided platelet count into four groups as non-thrombocytopenia (platelet ≥200,000 cell/µl), mild thrombocytopenia (platelet ≤200,000 cell/µl), moderate thrombocytopenia (platelet ≤150,000 cell/µl), and severe thrombocytopenia (platelet ≤100,000 cell/µl). In this study, infection with *E. canis* and *A. platys* was not statistically associated with platelet counts (p>0.05) (Table-3). This finding supports previous studies which showed that the mean values of platelet numbers in *E. canis-* and *A. platys*-infected dogs were not statistically different from non-infected dogs (p>0.05) [25,26].

**Table-3:** Association among *E. canis, A. platys*, and coinfections with platelet count.

Pathogens	Status	Platelet count (cell/µl) n (%)				Total n (%)	p-value	Average platelet count value (%)	95% CI

		>200,000	<200,000	<150,000	<100,000				
*E. canis*	+Ve	4 (5.9)	2 (2.9)	3 (4.4)	8 (11.8)	17 (25)	0.806	135,971	89,726-182,215
	−Ve	18 (26.5)	6 (8.8)	6 (8.8)	21 (30.9)	51 (75)	-	169,436	130,523-208,349
*A. platys*	+Ve	5 (7.4)	2 (2.9)	3 (4.4)	10 (14.7)	20 (29.4)	0.807	144,363	94,862-193,862
	−Ve	17 (25)	6 (6.6)	6 (6.6)	19 (27.9)	48 (70.6)	-	168,031	128,439-207,623
Coinfection	+Ve	2 (2.9)	1 (1.5)	1 (1.5)	4 (5.9)	8 (11.8)	0.965	142,313	74,329-210,295
	−Ve	20 (29.4)	7 (10.3)	8 (11.8)	25 (36.8)	60 (88.2)	-	163,571	129,017-198,125
Total		22 (32.4)	8 (11.8)	9 (13.2)	29 (42.6)	68 (100)			

+Ve=Positive; −Ve=Negative; *p<0.05, *A. platys=Anaplasma platys, E. canis=Ehrlichia canis*, CI=Confidence interval

## Conclusion

Tick-borne diseases cause mortality and morbidity in dogs worldwide. This study provides data on molecular detection of *A. platys* and *E. canis* in free-roaming dogs in Kalasin Province, Thailand, by nested PCR. In total, 29 of 68 (42.65%) dogs were infected with one or more pathogens. Tick vectors were found on 34/68 (50%) of dogs examined; however, the presence of ticks shows no significant associated with infections. Infection with *E. canis* and coinfection shows significantly correlated with PCV levels (p<0.05). Prediction of anaplasmosis and ehrlichiosis can be supported by hematological finding. Nevertheless, as the influencing by multifactor (such as infection by other agents, parasitemia, tissue damage, and responsibility of host immunity), anemia and thrombocytopenia cannot confirm the infection with *A. platys* or *E. canis*. However, data of hematological finding, especially in the naturally infected dogs living in the rural area, are still inadequate and are necessity for detailed in epidemiology surveys. This study is the first report of *E. canis* and *A. platys* infection in dogs in Kalasin Province, Thailand. In part of hematological response showed a significant relationship between *E. canis* infection and coinfection group with low levels of PCV, this finding strongly supported the previous studies that *E. canis* infection associated with severe anemia in dogs.

## Authors’ Contributions

SP conceived the project, design of the experiments and analysis of data, wrote the manuscript, reviewed, and edited the manuscript. PS, PC, WH, and BS collected the samples, performed the examinations, designed the experiment, and analysis of data. All authors read and approved the final manuscript.
